# Controlled-Release Phosphorus Fertilizers Manufactured with Chitosan Derivatives: An Effective Alternative for Enhanced Plant Development

**DOI:** 10.3390/plants14040610

**Published:** 2025-02-18

**Authors:** Eva García-Ilizaliturri, Enrique Ibarra-Laclette, Nicolaza Pariona-Mendoza, Carlos Espinoza-González, Antonio Cárdenas-Flores, José Humberto Valenzuela-Soto, Alan Josué Pérez-Lira, Claudia-Anahí Pérez-Torres

**Affiliations:** 1 Red de Estudios Moleculares Avanzados (REMAv), Instituto de Ecología, A.C. (INECOL), Xalapa 91073, Veracruz, Mexico; eva.garcia@posgrado.ecologia.edu.mx (E.G.-I.); enrique.ibarra@inecol.mx (E.I.-L.); nicolaza.pariona@inecol.mx (N.P.-M.); ljosuelira@gmail.com (A.J.P.-L.); 2 Departamento de Materiales Avanzados, Centro de Investigación en Química Aplicada (CIQA), Saltillo 25294, Coahuila, Mexico; carlos.espinoza@ciqa.edu.mx; 3 Departamento de Biociencias y Agrotecnología, Centro de Investigación en Química Aplicada (CIQA), Saltillo 25294, Coahuila, Mexico; antonio.cardenas@ciqa.edu.mx (A.C.-F.); humberto.valenzuela@ciqa.edu.mx (J.H.V.-S.); 4 Investigador por México-SECIHTI (before CONAHCyT) en el Instituto de Ecología, A.C. (INECOL), Xalapa 91073, Veracruz, Mexico

**Keywords:** encapsulated fertilizers, phosphate starvation, enhanced plant growth, high-frequency ultrasound, *Arabidopsis thaliana*

## Abstract

In modern agriculture, fertilizers are commonly used to increase crop yields; however, their negligent use can lead to environmental pollution and the waste of essential nutrients such as inorganic phosphate (Pi). Encapsulated fertilizers are a feasible alternative that could prevent these issues, as they can protect Pi from leaching and extend the interval between applications. In this study, we developed and tested innovative fertilizers (IFs) manufactured with KH_2_PO_4_, encapsulated with chitosan modified via high-frequency ultrasound treatment. The characterization of these fertilizers consisted of Fourier-transform infrared spectroscopy analysis and scanning transmission electron microscopy to determine their sizes and forms. In addition, we evaluated the phosphate release profile using electrical conductivity. The IFs were spheroidal microcapsules with an average diameter of 0.5–2 μM and showed slow-release behavior. Their efficacy was assessed via in vivo and in vitro assays, using *Arabidopsis thaliana* as a study model. As expected, the IFs promoted the growth of seedlings. One of the IFs showed enhanced growth promotion, contrasting with the control. This phenotype was likely promoted by this fertilizer due to the synergistic effect of Pi and the modified chitosan used as an encapsulant matrix. Our results highlight the potential of these formulations, which have unique properties and could be used on a large scale.

## 1. Introduction

At present, the increasing demand for food, the poor content of soil nutrients, and the reduction in arable areas threaten the food security of future generations [1]. While phosphorus (P) may be present in significant amounts within soils, the phosphate ion (Pi), which is the main chemical form that plants can assimilate, is scarce; this is mainly because phosphate anions react with cations in the environment to form insoluble precipitates [2,3]. P is an essential element for all life forms, and it is needed in constant supply to sustain optimal crop production; however, its low utilization/uptake rates on crops and its excessive use have serious environmental consequences, such as surface water eutrophication [4].

The nutritional stress caused by Pi starvation is manifested through changes in certain physiological traits that occur in the plant to cope with the deficiency in this macronutrient. Among these changes is a simultaneous reduction in shoot growth and increase in root proliferation, i.e., the formation of a highly branched root system, associated with a reduced primary root length, increased lateral root number and density, and increased frequency and length of root hairs [5,6,7]. These changes enhance the exploratory capacity of the roots to search for Pi-rich zones present in the soil [6,8]. Moreover, as part of this adaptative response, plants accumulate anthocyanin (mainly in the leaves) and increase the synthesis and secretion of organic acids into the rhizosphere to obtain Pi from insoluble inorganic compounds [9,10]. The release of phosphatases also represents a P-mining strategy that mobilizes organic P [11]. Arbuscular mycorrhizal fungi (AMF), which form a symbiotic relationship with the roots of over 80% of terrestrial plants, expand the absorptive surface and also contribute to phosphorus uptake by enabling solubilization and facilitating its transfer [12,13]. It is worth mentioning that plants can take up the available Pi via proteins located in the plasma membrane. These phosphate transporters can be of high or low affinity and are quickly induced under Pi deficiency. The changes described above prioritize the maintenance of phosphate homeostasis, but a prolonged lack of Pi eventually leads to biomass loss [14,15].

Fertilizers are essential in modern agriculture, serving to enhance plant growth and increase crop quality and production. However, their manufacturing faces challenges because there is an increasing demand to develop highly effective products that require less frequent application and to reduce or avoid adverse ecological effects [16]. In response to this need, recent research has focused on developing fertilizers whose active agents are encapsulated. In this way, it is not only protected but is gradually released, thus reducing its application to the minimum amount required. Generally, the coating layer is poorly soluble in water, allowing slow release while avoiding the direct interaction of the active agent (e.g., Pi) with biotic and abiotic factors in the soil [17]. Synthetic polymers have been widely used in this context because their larger molecular structures allow them to form complex networks; however, natural polymers and waxes are rapidly gaining attention because they can also efficiently encapsulate active agents and are environmentally friendly [16,18].

Due to properties such as their low cost, availability, biodegradability, biocompatibility, and lack of or minimal toxicity, natural polymers such as chitosan, candelilla wax, sodium alginate, starch and its derivatives, cellulose and its derivatives, lignin, agricultural residues, biochar, and polydopamine have been successfully used as encapsulating coating layers to generate gradual- or controlled-release fertilizers [19,20,21,22,23,24]. Some of these, such as chitosan, have been used in agriculture as biostimulants, which promote plant growth and induce biotic stress tolerance, among other beneficial effects. For example, there is evidence that the exogenous application of chitosan in commercially important species, such as tomato, watermelon, and coffee, increases both the absorption of macro- and micronutrients from the soil and the accumulated biomass of the fruit [25,26,27,28]. When applied to fruits and vegetables, it reduces transpiration, slows fruit ripening, and prevents postharvest decay and diseases caused by fungi and bacteria [29,30,31,32]. Thus, besides being environmentally friendly, the advantages of fertilizers that contain chitosan as an encapsulant matrix are associated with its low solubility and its capacity to act as a biostimulant [33]. However, several methodologies can be applied to modify chitosan so that properties such as the delayed release of the active compounds or plant response induction can be improved, resulting in additional benefits in some cases [34].

In the present study, we aimed to develop distinct innovative fertilizers (IFs) using modified chitosan as a coating to control Pi release. We hypothesized that, in contrast with the control (monobasic potassium phosphate (KH_2_PO_4_), used as a conventional fertilizer), our manufactured IFs would exhibit improved activity to promote plant growth beyond conventional fertilizers. This is because they can slow the release of the phosphate salts, and it is also due to the biostimulant properties of the encapsulant matrix (modified chitosan). Thus, after manufacturing three different IFs, we characterized them using Fourier transform infrared (FTIR) spectroscopy and scanning electron microscopy (SEM). The phosphate release rate of these fertilizers was determined via electrical conductivity tests. Finally, the effectiveness of the manufactured IFs was confirmed through in vitro and in vivo assays, in which the growth and development of *Arabidopsis thaliana* (L.) Heynh seedlings were evaluated.

## 2. Results and Discussion

### 2.1. Encapsulation of KH_2_PO_4_ Using Modified Chitosan to Obtain Innovative Fertilizers

**Figure 1:**
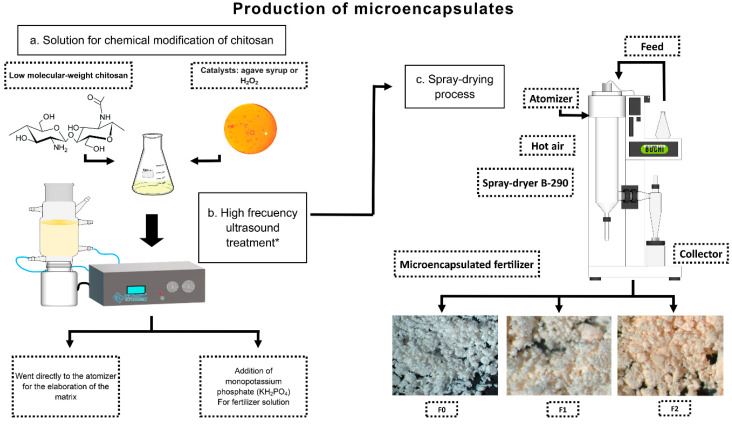
Schematic illustration of the synthesis/formulation of innovative phosphate fertilizers coated with chitosan (F0) or chitosan modified via ultrasonic treatment (F1 and F2). The asterisks in the boxes indicate steps not performed in the F0 fertilizer manufacturing process, such as modifying the encapsulant matrix.

Several physical, chemical, and biological methods can be used to modify chitosan [35]. With improved performance through modification, the application of chitosan-based materials can be expanded to different fields, such as water treatment, food processing, pharmaceuticals/biomedicine, textiles, and—as demonstrated in this study—agriculture. Figure 1 shows a schematic representation of the workflow, and the methodology used (now patented [36]), illustrating the modifications made to the chitosan via high-frequency ultrasound irradiation to generate an encapsulant matrix and the implementation of the spray drying technique for fertilizer encapsulation.

Henceforth, we refer to the encapsulant matrices and the innovative fertilizers manufactured with them as follows: the non-modified chitosan or control encapsulant matrix is denoted as CM0 (where CM—chitosan matrix); the modified chitosan encapsulant matrices are referred to as UMCM1 and UMCM2, depending on the type of additive used to promote modification. The IFs manufactured using these encapsulant matrices (i.e., KH_2_PO_4_ encapsulated with CM0, UMCM1, or UMCM2) are denoted as F0, F1, and F2, respectively (see Section 3 for details or [36] for any additional technical aspects related to the manufacturing).

### 2.2. Innovative KH_2_PO_4_–Chitosan-Based Fertilizers Consisting of Heterogeneous Microparticles

**Figure 2:**
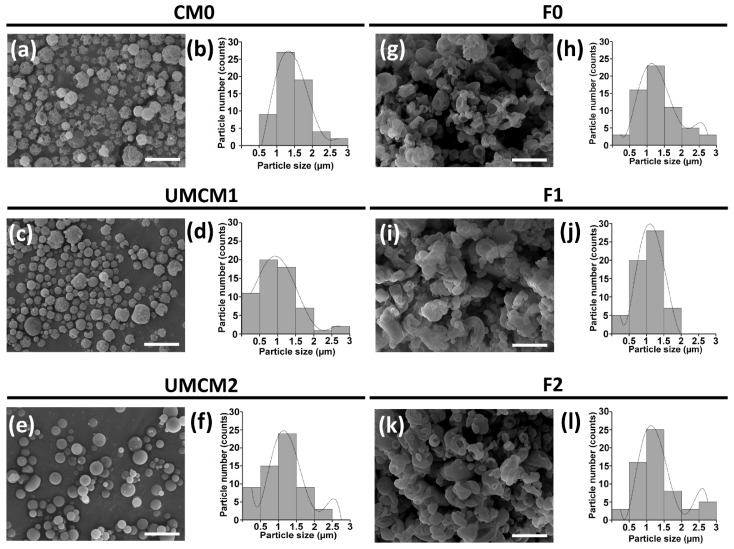
Morphological characterization of the innovative fertilizers and the encapsulant matrices used to generate them. SEM images (**a**,**c**,**e**,**g**,**i**,**k**) and histograms of the diameter ranges of the microspheres formed with both the encapsulant matrix (chitosan, CM0, and modified chitosan, UMCM1 and UMCM2; (**b**,**d**,**f**)) and innovative phosphate fertilizers (F0, F1, and F2; (**h**,**j**,**l**), respectively). Scale bar = 5 µm; 5000× magnification.

SEM analyses allowed us to observe the morphologies of the modified polymers and the encapsulated fertilizers. As shown in Figure 2a,b (respectively), the micrographs of CM0 indicated particles with uneven surfaces due to the presence of some cavities, with a size ranging from 1 to 2 µm. Regarding the encapsulant matrix UMCM1, a spheroid shape with greater uniformity in size was observed; moreover, particles with a size of around 1–1.5 µm were predominant (Figure 2c,d). Finally, the encapsulant matrix UMCM2 presented a well-defined spherical shape, with slight roughness and minor pores on the surface (Figure 2e,f). A clear tendency to generate aggregates was observed, while the size of these particles ranged from 0.5 to 1.5 µm. Meanwhile, the morphologies of fertilizers F0, F1, and F3 (Figure 2g,i,k) consisted of aggregates of particles with a rough surface and folds, with some isolated particles with heterogeneous sizes (0.5–2 µm; Figure 2h,j,l). It has been shown that the integrity of particles and the differences in their morphologies are in part determined by the osmolarity of the stock solution. Thus, at a greater proportion of phosphate ions, the particle experiences a reduction in turgor; meanwhile, in the absence of ions, the matrices implode, forming folds [37]. On the other hand, the morphology of microparticles (microencapsulated) obtained via spray drying is influenced by the interactions between the solute and the solvent [38]. These interactions, which play a crucial role in the drying process, are largely determined by the distribution of functional polar groups in the solute that can interact with the solvent. As discussed earlier, high-frequency ultrasound can generate conformational changes in polymers, altering or modifying the functional groups [39]. In this study, the microencapsulates were analyzed via FTIR to investigate these assumptions.

### 2.3. Differences in Innovative Fertilizers and Encapsulant Matrices According to FTIR

**Figure 3:**
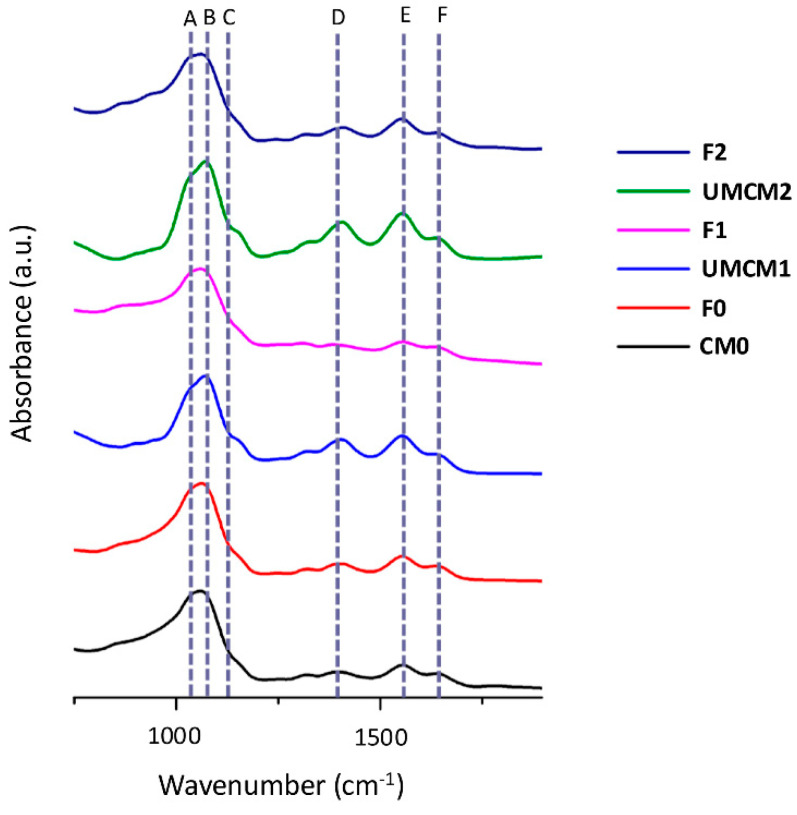
Comparison of the FTIR spectra of the innovative fertilizers (F0, F1, and F2) and the encapsulant matrices used to generate them (CM0, UMCM1, and UMCM2, respectively). The dotted lines (A–F) indicate the six characteristic maximal absorption bands of some functional groups of chitosan.

The FTIR spectra of the samples displayed absorption bands that are commonly used to identify the characteristic functional groups of chitosan. For example, the presence of residual N-acetyl groups was confirmed by the bands at around 1637 cm^−1^ (C=O stretching of amide I) and 1325 cm^−1^ (C–N stretching of amide III); in Figure 3, these are absorption bands F and D, respectively. The band at 1559 cm^−1^ (E) corresponds to the N–H bending of the secondary amine [40,41]. The CH_2_ bending and CH_3_ symmetrical deformations were confirmed by the presence of bands at around 1423 and 1375 cm^−1^, respectively. The absorption band at 1153 cm^−1^ (C) could be attributed to the asymmetric stretching of the C–O–C bridge, while the bands at 1066 and 1028 cm^−1^ (in Figure 3, bands A and B, respectively) correspond to C–O stretching. Other authors described the presence of these characteristic bands in the spectra of chitosan and even in some spectra derived from modified chitosan [42,43,44,45,46]. As expected, regardless of the modifications that took place after ultrasound treatment (and independently of the addition of inorganic components or changes in the type of alkali used for neutralization; see Section 3), these six characteristic absorption bands of chitosan were found in the obtained FTIR spectra of the IFs (F0, F1, and F2) and the encapsulant matrices used to generate them (CM0, UMCM1, and UMCM2) (Figure 3). It should be noted that both ultrasonic-modified chitosan matrices (UMCM1 and UMCM2) showed a larger number of amide-type bonds (in Figure 3, absorption bands D, E, and F, respectively). This finding could explain the differences observed in the morphologies of these microparticles. When using spray drying [38], the morphologies of microparticles may depend on the number of functional polar groups that interact with the solvent (water). Functional polar groups, such as hydroxyl, carboxyl, or amino groups, can form hydrogen bonds with water molecules, which affect the solubility and diffusion of the solutes. The larger the number of functional polar groups, the higher the solute’s affinity for water and the lower the surface enrichment during droplet evaporation. This may result in smoother, more homogeneous, and denser particles. Conversely, the smaller the number of functional polar groups, the lower the solute’s affinity for water and the higher the surface enrichment during droplet evaporation. This may result in more heterogeneous, collapsed, and porous particles.

Because the interactions between the functional polar groups of the polymer matrices and the solvent (water) are altered due to conformational changes after sonication, the phosphate release behavior can differ for each polymer matrix. These interactions could explain, at least in part, why the chitosan-modified products (F1 and F2) were more elastic and absorbent than the fertilizer formulated with conventional chitosan (F0) (see below).

### 2.4. Gradual Release of KH_2_PO_4_ Content

**Figure 4:**
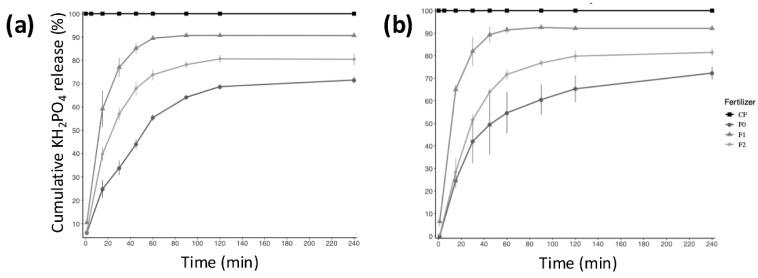
Pi release kinetics in water at pH 6.0 (**a**) and 4.0 (**b**) for the innovative fertilizers, i.e., microspheres in which Pi is encapsulated by chitosan or ultrasonic-modified chitosan (formulations F0, F1, and F2, respectively). The release kinetics of the conventional fertilizer (CF) are also shown.

As electrical conductivity measurement is a common method used to determine the ion content dissolved in water [47,48,49,50], this was used to determine the P release rate from the dialysis-derived solution (see Section 3 for details). F0 was the fertilizer that showed the slowest release. The conductivity in the resulting solution showed a continuous increase over time due to the release of the active compound (KH_2_PO_4_), reaching a maximum of 70% release after 240 min (Figure 4). This finding suggests that 30% of the active compound was retained in the F0 fertilizer, even when the release assays were performed in an aqueous solution. This was probably due to the mechanism of release from the encapsulant matrix, which was a hydrophilic polymer (CM0 or unmodified chitosan). F1 and F2 also showed continuous release, but, in contrast with F0, these formulations released around 80 and 90% of the active compound, respectively (Figure 4). Despite the differences in the release rate, these three formulations (F0, F1, and F2) could be considered controlled-release fertilizers because they possessed the essential features required to be considered as such; in particular, they gradually released the active compound, prolonged its lifetime, and could potentially reduce the number of applications required [51]. It should be noted that the Pi release rate across time was almost identical and was independent of the pH in the solution used in our release assays (deionized water at pH 6 and 4; see Figure 4a,b, respectively). This property is noteworthy as it suggests that our IFs could be used in different soils or substrates with a wide pH range. Chitosan is the only polycation in nature, and its charge density depends on the degree of acetylation and the pH of the media. The solubility of the polymer depends on the degree of acetylation and molecular weight (MW) [52]. Chitosan oligomers are soluble over a wide pH range, from acidic to basic values. In contrast, chitosan samples with a higher MW are only soluble in acidic aqueous media, even at high degrees of deacetylation. This is why a significant number of chitosan derivatives with enhanced solubility have been synthesized [53]. Our results suggest that the UMCM1 and UMCM2 matrices could efficiently encapsulate CF and avoid its precipitation, which would occur if KH_2_PO_4_ was applied in acidic soils without a coating or cover. It should be noted that, in some acidic soils, the predominance of aluminum (Al) and iron (Fe) oxides in both crystalline and amorphous forms reduces the solubility of inorganic P through fixation on positively charged surfaces and the formation of insoluble Al and Fe precipitates [54,55]. We acknowledge that further experiments must be conducted in future work, but these results confirm that newly formulated IFs represent a promising development.

To understand the mechanics behind the release of the active compound, the measured Pi release rates (see above) were compared with those obtained using mathematical models that are commonly used to predict drugs’ overall release behavior and delivery. These mathematical models were as follows: (i) the order 0 model, which is equal to instant release [56]; (ii) Higuchi’s empirical model, which is used for encapsulated core-type systems with single-dimension diffusion [57]; and (iii) the Korsmeyer–Peppas model, which is a practical model that describes the release according to the structural characteristics of polymeric membranes that can absorb moisture until an equilibrium is reached with the medium, considering experimental variables such as the solute size, the ability of the polymer to expand, and the mesh size [58]. It should be noted that the release kinetics are determined by the encapsulant polymer’s hydrophobicity and dissolution capacity; these depend on their molecular weight, end groups, and composition, among other factors [59]. Although many models exist that can be used to study or describe the release profile of an encapsulated active compound, those mentioned above are the only ones that are applicable to our IFs. This is because they consider the period in which the kinetic release was performed, as well as because chitosan and modified chitosan are biodegradable and biocompatible polymers but with low water solubility. Therefore, only models in which the active compound can diffuse through the polymer can be used [59]. It should also be noted that the models used in this study have been previously used to predict drugs’ overall release behavior when encapsulated with chitosan [60]. The model that best fits the release data is determined via the coefficient of determination (R^2^), which is influenced by the effects of the formulation parameters [59].

Table 1 shows the equation used in each release model, the considered parameters, and the resulting coefficient of determination. Although the correlation levels are high, we could rule out the zero-order release system since innovative fertilizers’ release rate does not resemble a straight line parallel to the X-axis (Figure 4). In the case of the Higuchi model, the weak correlation may have been due to the fact that the encapsulation system was not of the “core” type; rather, it was an interleaved and three-dimensional system. The Korsmeyer–Peppas model not only showed strong correlation values but also reflected some of the main characteristics of the encapsulating matrices (chitosan or modified chitosan), such as low solubility, minimal particle erosion, and the tendency to swell [59]. Consequently, it could be inferred that the release of phosphate in the particles was dependent on the nature of their interactions with the functional polar groups of the polymer matrix and the water in the medium, as the matrices used to encapsulate the phosphate released their content when the polymeric chains opened and a balance with the environment was achieved.

In this context, Laucirica et al. developed and studied the interactions between amines and phosphate ions in solid-state nanostructures, finding that the phosphate binding was higher compared with that of other divalent anions, and it took place even in electrostatically hindered conditions [61]. These findings reinforce the idea that the amine–phosphate interactions are crucial for the self-assembly of nanostructures, as they determine the molecules’ binding behavior and the effects of nanoconfinement. These interactions could also influence the morphologies of phosphate fertilizers encapsulated via spray drying into polymer matrices containing amine functional groups. The amine groups on the polymer’s surface could form strong hydrogen bonds with the phosphate groups, leading to the higher affinity of the solute for water and lower surface enrichment during droplet evaporation. As a result, the microparticles produced would be smoother, more homogeneous, and denser. The nanoconfinement effects could affect the fertilizer’s crystallization and aggregation into microparticles, resulting in different shapes and sizes.

Similarly, the nature of the amine–phosphate interactions could explain the differences in the release behavior of phosphate ions in an aqueous medium. According to Laucirica et al., the binding of phosphate anions to amine functional groups is a specific and strong interaction that depends on the pH and the phosphate concentration; therefore, we could state that phosphate release depends on interactions between the chemical groups of chitosan and water, serving to promote the swelling of the particles due to its penetration. This causes the distension of the chains, reordering (swelling/relaxation), and, consequently, the solubilization of the phosphate fertilizer, followed by diffusion towards a less concentrated surrounding solution.

It should be noted that, although these release models mainly reflect the possible release scenarios of encapsulated compounds in an aqueous solution and do not consider the dynamic nature of the soil properties, they have frequently been used to determine the release mechanisms of controlled- or slow-release fertilizers in which inorganic or organic polymers (including chitosan) are used as coatings [62,63,64]. Moreover, a recent study that evaluated the biodegradation and viability of some chitosan-based microencapsulated fertilizers showed that, while the fertilizer release was much faster in water than in soil, approximately one hour of release in water was equal to 20 days in soil [47]. This observation is highly consistent with our results, which are presented below.

### 2.5. Controlled and Slow Release in Soil

**Figure 5:**
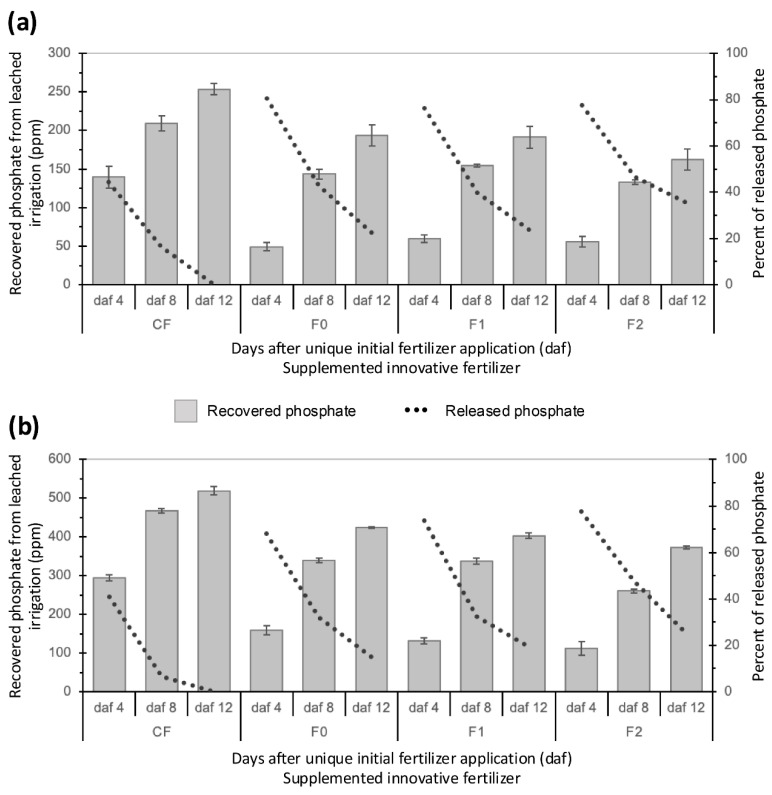
Pi released from innovative fertilizers into the sand–perlite–vermiculite substrate. The bars in the plots indicate the amount of released Pi recovered from the leached water when the fertilizers were supplemented at 250 and 500 ppm ((**a**,**b**), respectively). The dotted lines represent the retained Pi in the encapsulated fertilizers over time once the soil reached the maximum water-holding capacity (MWHC).

Considering that the release rate depends on the properties of the coating (i.e., the chitosan-modified matrix) and the ionic gradient difference between the fertilizer and the surrounding environment, assays were performed to illustrate the slow-release (controlled-release) behavior of the IFs in soil. In this work, as “soil”, we used a substrate composed of a mixture of sand, perlite, and vermiculite (see Section 3). This chemically inert substrate allowed us “to mimic” (and control) the water-holding capacity and evaluate the effect of supplying (or releasing) nutrients (Pi) in a controlled manner [65]. Independently of the amount supplemented (either 250 or 500 ppm), after four days, the sand–perlite–vermiculite substrate retained 40% of the CF at the maximum water-holding capacity (MWHC); the remaining (60%) was found in the leached water (Figure 5a,b). In contrast, the IFs released only around 22% of the encapsulated Pi. After eight days, the CF in the substrate was almost exhausted, i.e., 90% of the supplemented Pi was found in the leached water, while the IFs retained 35 to 45% of the Pi in the substrate (Figure 5). Notably, despite maintaining the soil for 12 days at the MWHC, the IFs retained 17 to 30% of the encapsulated Pi (Figure 5), with F2 being the fertilizer that retained the largest amount of Pi due to its slower release rate. These results were consistent with those obtained in the release assay performed in water (see above). Moreover, it was concluded that, when the soil was maintained at the MWCH, the F1 and F0 fertilizers released around 9% of the encapsulated Pi daily, while F2 released only 6.5%.

It should be noted that the IFs, in contrast to the CF, did not show the immediate release of Pi when they encountered water or when the surrounding environment reached the maximum water-holding capacity. This initial retention occurred because the particles could resist the osmotic pressure, avoiding the immediate diffusion of the Pi [66,67]. This behavior observed in the release assays coincided with the proposed release model, in which the humidity of the surrounding medium is predicted to cause the gradual swelling of the polymeric chains of the encapsulant matrix, promoting the prolonged release of the Pi via diffusion [68].

### 2.6. Responses of Arabidopsis thaliana Seedlings to Innovative Fertilizers

**Figure 6:**
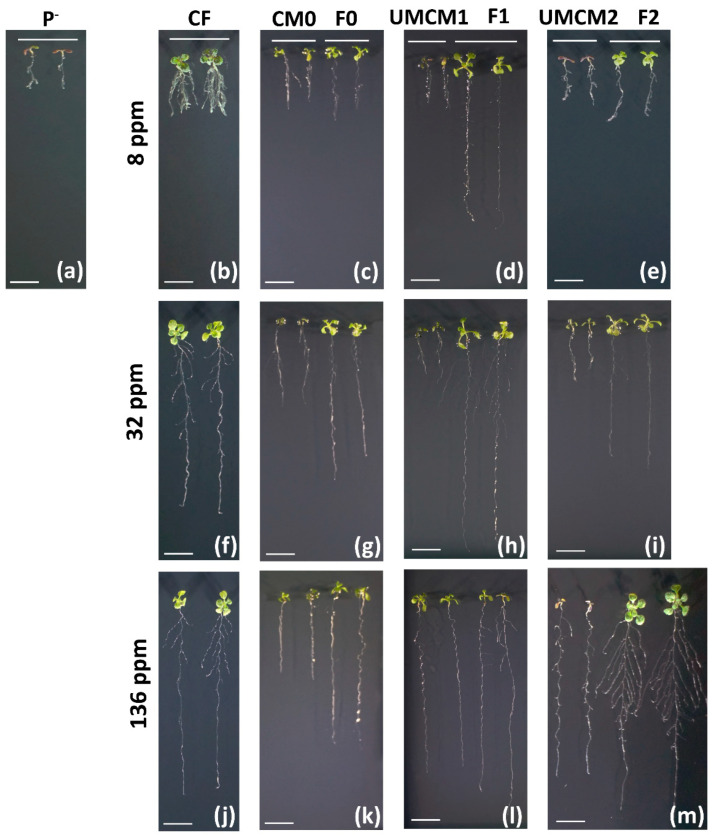
In vitro assay of *Arabidopsis thaliana* growth and development under treatment with innovative fertilizers. Fifteen-day-old seedings were germinated in MS medium supplemented with the innovative phosphate fertilizers (F0, F1, and F2) or the encapsulant matrices used to formulate them (CM0, UMCM1, and UMCM2, respectively) (**c**–**e**,**g**–**i**,**k**–**m**). As a negative control, *A. thaliana* seedlings were germinated and grown under phosphate-limited conditions (P^−^; (**a**)). The positive control seedlings were supplemented with conventional fertilizer (CF; (**b**,**f**,**j**)). Note that all seedlings that were grown in supplemented media were grown at different concentrations (8, 32, or 136 ppm) of either a fertilizer (CF, F0, F1, or F2) or a chitosan-encapsulated matrix (CM0, UMCM1, or UMCM2). The photographs are representative of at least 15 plants analyzed. Scale bar = 1 cm.

To determine the effect of the IFs, *A. thaliana* (Col-0) seeds were germinated in vitro using vertically oriented Petri dishes containing solid MS media supplemented with different concentrations of Pi (8, 32, or 136 ppm). These concentrations of Pi were administered to the seedlings either through the addition of a conventional fertilizer (CF) or through the independent addition of each formulation (F0, F1, and F2). As a negative control, *A. thaliana* seeds were also germinated in a medium lacking a Pi source. Moreover, to evaluate the possible effect of the encapsulant matrix (CM0, UMCM1, and UMCM2) on the growth of the *A. thaliana* seedlings, each matrix was also independently added to the culture medium (see Section 3 for details). As expected, the *A. thaliana* seedlings that were germinated on the medium without a phosphate source showed the distinctive and well-described Pi starvation phenotype, i.e., a short primary root with a high density of lateral roots (LRs) and abundant root hairs [5,7] (Figure 6a). It should be noted that the tender shoots in such seedlings turn purple due to the accumulation of anthocyanin, flavonoids, and condensed tannins [10]. As the amount of CF was increased (8, 32, or 136 ppm), the *A. thaliana* plants gradually recovered to the Pi-sufficient phenotype, with the marked growth of the primary roots and shoots.

All IFs at concentrations as low as 8 ppm showed the longest primary roots, in contrast with the CF; this difference was particularly significant for the F1 fertilizer (Figure 6b–e). The F2 fertilizer required a higher concentration (32 ppm) to show a similar effect on the primary root length (Figure 6i). Meanwhile, the F0 fertilizer, which showed the slowest KH_2_PO_4_ release kinetics among the studied formulations, was associated with the most delayed growth. Even at the highest concentration (136 ppm), primary root development was not induced at the same level as in the control plants when they were grown with similar concentrations of the conventional fertilizer (Figure 6j,k). Interestingly, the matrices alone (CM0, UMCM1, and UMCM2) affected primary root growth. This growth promotion was more evident at the higher concentration (136 ppm). Surprisingly, the primary root lengths of plants grown with only the chitosan-modified encapsulant matrices (UMCM1 and UMCM2) were similar to those seen in the control conditions (136 ppm of CF), despite the fact that these treatments did not contain a Pi source (Figure 6j,l,m). The observed differences in the primary root length due to the used treatments (either the encapsulant matrices CM0, UMCM1, or UMCM2 or the fertilizers CF, F0, F1, or F2), as well as the differences observed in the number of lateral roots, were mostly significant (Figure S1). This improvement in the growth of the root system has also been reported in species such as maize and grapevine, in which chitosan induced the rooting of cuttings or promoted the elongation of the primary roots and lateral root formation [69,70].

In contrast to the CF, F2 at 136 ppm showed the best effect on the growth of *A. thaliana*. In this formulation, a growth-promoting effect was synergistically induced by the chitosan-modified encapsulant matrix (UCMCM2) and the active compound (KH_2_PO_4_) (Figure 6m). *A. thaliana* (as with other angiosperm plants) can recognize chitin and chitosan through specific protein receptors [71]. These exogenous elicitors (chitin and chitosan) are recognized as pathogen-associated molecular patterns (PAMPs) and trigger immune-related defense responses [72]. Recently, it has been demonstrated that the perception of these and other exogenous elicitors, when recognized without pathogen pressure, promotes plant growth (as reviewed in [73]). It has also been demonstrated that the recognition of chitin and chitosan depends on their polymerization and degree of acetylation [74]. This could explain, at least in part, the distinct plant responses observed in each of the different treatments (either CM0, UMCM1, or UMCM2 or F0, F1, or F2). Based on the presented results, it can be observed that the F2 fertilizer almost completely reversed the Pi starvation phenotype at concentrations as low as 32 ppm (four times lower than optimal). When this fertilizer was used at 136 ppm (the highest concentration tested), the plant’s growth was significantly promoted, increasing the total size by up to 70, i.e., larger shoots and a highly branching and proliferating root system. This is consistent with previous reports that have indicated that foliar chitosan applications can increase the yields of species of commercial interest, such as potatoes, strawberries, and tomatoes, by up to 60% [28,75,76].

### 2.7. GUS Activity in AtPT2::uidA Transgenic Line in Line with KH_2_PO_4_ Release Profile of Innovative Fertilizer

**Figure 7:**
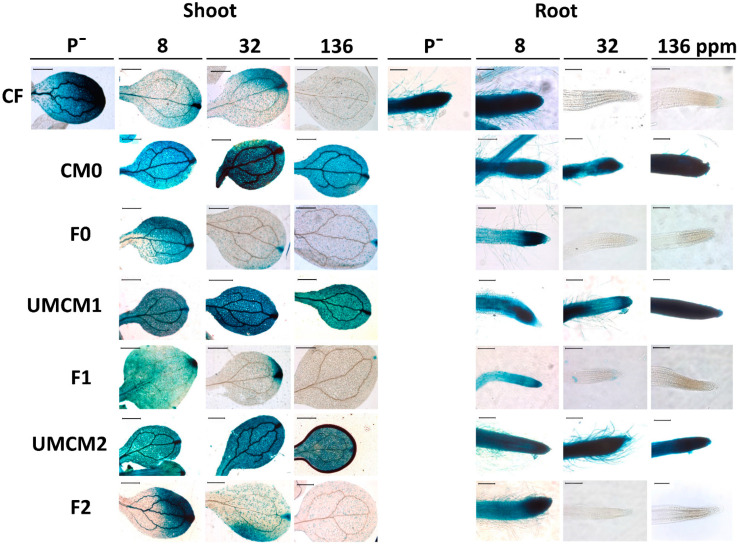
Effects of the innovative fertilizers (F0, F1, and F2) and the encapsulant matrices used to formulate them (CM0, UMCM1, and UMCM2) on the expression of the *uidA* gene in the *AtPT2:uidA A. thaliana* transgenic line. As in the assay performed with the *A. thaliana* wild-type genotype (Figure 6), negative (P^−^) and positive controls (CF) were also included. The concentrations tested were 8, 32, and 136 ppm of either a fertilizer (CF, F0, F1, or F2) or a chitosan encapsulant matrix (CM0, UMCM1, or UMCM2) added to the culture medium. Histochemical staining of the GUS activity in these 15-day-old *AtPT2:uidA* seedlings was carried out according to the protocols described in the Section 3. The photographs are representative of at least 20 seedlings analyzed. Scale bar = 500 μM (shoot) and 100 μM (root).

AtPT2 is a high-affinity phosphate transporter that quickly increases its expression under Pi starvation conditions to improve the uptake of this essential macronutrient. Therefore, *AtPT2::uidA* transgenic seedlings show high GUS activity when the Pi supply is limited. In contrast, when the Pi supply is optimal or gradually increases, the GUS activity tends to decrease or disappear. As expected, the GUS activity was induced in the shoots and roots in *AtPT2::uidA* transgenic seedlings that were germinated and grown without a phosphate source (P^−^ treatment; see Figure 7). Interestingly, when Pi was supplied via the CF or any of the IFs (F0, F1, and F2), the GUS activity decreased in a dose-dependent manner (Figure 7 and Figure S2). Notably, the GUS activity was consistent with the release profile obtained for each formulation, i.e., F0 was the fertilizer with the slowest release rate, followed by F2 and F1, respectively (Figure 4, Figure 5, Figure 6 and Figure 7). Interestingly, *AtPT2::uidA* transgenic seedlings that were germinated and grown with the CM0, UMCM1, and UMCM2 encapsulant matrices but without a source of phosphate showed a GUS activity pattern that was highly similar to that observed in the treatment without Pi (Figure S2). These results can be considered as additional evidence that the partial restoration of root growth promoted by the encapsulant matrices is due to a growth promotion effect that acts mainly on the primary root. Moreover, compared with the use of chitosan, this promoting effect was improved following the modifications caused by the ultrasonic treatments.

Considering the plant phenotypes observed in the in vitro bioassays described above, our results demonstrate that the IFs (F0, F1, and F2) promoted changes in the growth and development of the *A. thaliana* seedlings. These changes were most likely caused by both the concentration of Pi and the growth-promoting effect of the modified chitosan, which acted synergistically; this effect was induced by the encapsulant matrix used to formulate each of the IFs.

### 2.8. Enhancements in Growth and Development of Arabidopsis thaliana in In Vivo Bioassays with F2 Controlled-Release Fertilizer

**Figure 8:**
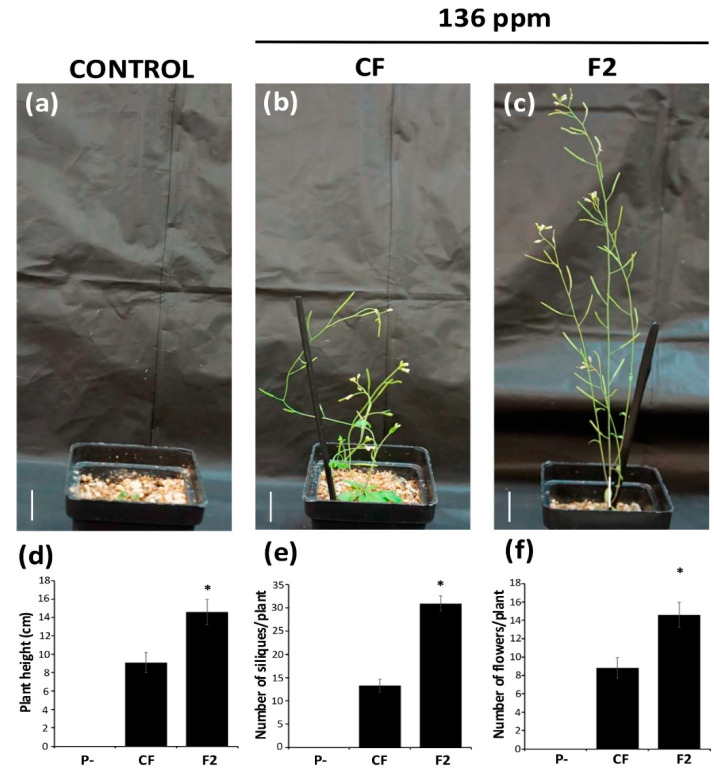
Effects of F2 fertilizer on *A. thaliana* plant growth when using an inert substrate under greenhouse conditions. Here, 21-day-old seedlings, which were first germinated and grown in an in vitro system under optimal phosphate conditions (136 ppm), were transferred to an inert sterile sand–perlite–vermiculite (2:1:1) substrate without a fertilizer (control) or supplemented with either the CF or F2 fertilizer. The photographs show the rosette size after 15 days of growth under each treatment ((**a**,**b**,**c**), respectively). Mature plants grown on the same substrates for 30 days are also shown (**d**–**f**). At least 10 plants for each treatment were used as replicates, but the photographs show those plants selected as representative. Scale bar = 1.5 cm. In the case of mature plants, the inflorescent height was measured, and the number of flowers and siliques was determined (**d**–**f**). Asterisks by the bars indicate a significant difference (*p* ≤ 0.05).

Based on the results of the in vitro assays and considering that the F2 fertilizer was identified as the most promising, an in vivo bioassay was carried out in which *A. thaliana* plants were grown in a sand–perlite–vermiculite substrate and under greenhouse conditions. The “soil” (or inert substrate), the irrigation frequency, and the greenhouse conditions are described in detail in the Section 3. As expected, in line with the in vitro results, plants fertilized with the innovative fertilizer F2 showed enhanced growth compared to the unfertilized *A. thaliana* plants or those fertilized with the CF. The growth-promoting effect of the F2 fertilizer was clearly visible. This does not only involve an increase in the rosette size (Figure 8a–c) but is also manifested in the height of the inflorescences of mature plants (Figure 8d) and by a greater number of flowers and siliques (Figure 8e,f, respectively). These results confirm that the F2 fertilizer can induce both vegetative and reproductive growth, highlighting its potential use not only as a controlled-release fertilizer but also as a treatment to significantly promote plants’ growth. It is able to reduce the amount of phosphorus fertilizer that is applied to the soil by three to four times. This could offer a novel approach in the design of smart fertilizers that respond to the soil conditions and the plant’s specific needs.

Despite the lack of related studies, we acknowledge that many environmental factors could influence the pace at which nutrients are released from coated or encapsulated fertilizers [77]. These factors, which include soil microbial activity, soil moisture, rainfall, and temperature [78,79,80], need to be addressed in future studies because they may influence crop growth if not appropriately managed. There are also clues to a possible influence of soil structure [81], organic matter [82], and changes in pH caused by organic acids produced by soil microorganisms [83].

While it is expected that controlled-release fertilizers encapsulated either with organic or inorganic polymers will contribute to modern, sustainable agriculture [84,85], it has been demonstrated that the prolonged use of controlled-release fertilizers, if coated with synthetic polymers, can result in the accumulation of undecomposed polymer capsules in the soil [86]. Such amounts of residual microplastics can significantly affect the soil’s ability to retain moisture, micro-elements, and bacteria, in turn affecting the amount of moisture consumed and the quality of the resulting crop [87,88]. In contrast, the encapsulation of active compounds in natural polymers is considered an “eco-friendly” next-generation strategy for sustainable agriculture and crop management [89]. Interestingly, biostimulant properties have been observed in some formulations in which active compounds are encapsulated with chitosan-based polymers, and these have been successfully applied in some crops [90].

## 3. Materials and Methods

### 3.1. Manufacture of Innovative Fertilizers (KH_2_PO_4_ + Chitosan-Modified Microencapsulates)

Commercial chitosan (Sigma-Aldrich, Darmstadt, Germany) with a low molecular weight (50 to 190 KDa) and monobasic potassium phosphate (KH_2_PO_4_; Sigma-Aldrich, St. Louis, MO, USA) were used as the encapsulant matrix and conventional fertilizer, respectively. Both the raw materials and the chemical reagents used to modify the chitosan, manufacture the IFs, and perform the bioassays were of analytical grade. The preparation of these fertilizers is innovative; as such, the method of modifying chitosan and generating new physicochemical properties, as well as the formulation of the fertilizers, was patented before the submission of this manuscript (U.S. Patent US11639319B2 by [36]. In this process, chitosan is exposed to high-frequency ultrasound waves (>500 kHz), which induce molecular rearrangement and prevent the rupture of the polymer chains, thus improving the polymer’s physicochemical properties, especially its solubility at a pH level > 7. The modified chitosan polymer was used to encapsulate the phosphate fertilizer (KH_2_PO_4_) via spray drying. Briefly, after vigorous stirring for 24 h and at room temperature, a 1% (*w*/*v*) chitosan solution (CS-soln.) was prepared by dissolving the low-weight chitosan powder in an aqueous solution of 1% (*v*/*v*) acetic acid (Merck, Darmstadt, Germany). The CS-soln. was filtered to remove impurities or residues. Then, three distinct encapsulant matrices were prepared: the first one consisted of the CS-soln. alone, i.e., without additives or ultrasonic modification. For the chitosan modifications, the CS-soln. was mixed with H_2_O_2_ (Sigma-Aldrich, Darmstadt, Germany) or with sugar-type maguey honey (Villa de Patos, General Cepeda, Mexico). Each resulting aqueous solution was subjected to high-frequency ultrasonic modification with a Meinhardt Multifrequency Generator (Meinhardt Ultrasonics Leipzig, Germany) using a sonotrode configured to operate at an ultrasonic frequency of 858 kHz or 1134 kHz, with ultrasound power of 60% and a temperature of 20 °C, for 30 min. These solutions were separately subjected to spray drying to obtain non-modified chitosan or the control encapsulant matrix (CM0; CM from Chitosan Matrix) and the ultrasonic-modified chitosan matrices (UMCM1 and UMCM2, respectively). It should be noted that the only difference between UMCM1 and UMCM2 was in the type of additive used to generate the CS-soln. (either H_2_O_2_ or sugar-type maguey). See our patent for details [36].

The three chitosan solutions described above were independently mixed with a KH_2_PO_4_ solution at a 1:1 ratio (*v*/*v*) to manufacture the IFs. These formulations were stirred at 40 °C for 30 min before being processed via spray drying. In the last step (spray drying), a Buchi mini spray dryer, model B-290, was used (BÜCHI Labortechnik^®^, Flawil, Switzerland). The spray drying conditions (previously tested) consisted of a liquid feed of 2–5 mL/min, an inlet air temperature of 160 °C, an outlet air temperature of 90 °C, a suction speed of 90% (31.5 UMCM1/h), an atomizing airflow of 80 L/h, and a 7 µm nozzle size (details in U.S. Patent US11639319B2 by [36]. The collected powder was stored at low humidity (20–40%) and room temperature. For practical purposes, the IFs (once dried and powdered) were named F0, F1, and F2 (each corresponding to the active agent (KH_2_PO_4_) encapsulated with CMO, UMCM1, or UMCM2, respectively).

### 3.2. Scanning Electron Microscopy

The morphologies and structures of the innovative microencapsulated fertilizers were analyzed using scanning electron microscopy (SEM). All fertilizers (F0, F1, and F2) and the encapsulating matrices (CM0, UMCM1, and UMCM2) were mounted in aluminum sample holders covered with conductive double-sided carbon adhesive tape. Later, they were coated with gold in a Quorum 150R metal ionizer (Philadelphia, PA, USA) prior to their observation and elemental microanalysis in a JEOL microscope, model JSM-IT300LV, adapted with an OXFORD EDS X-MaxN Silicon Drift Detector (Tokyo, Japan) working at 15 kV. The micrographs were captured at 5000× magnification. All image analyses, including particle size characterization, were performed using the free ImageJ software v1.54 [91].

### 3.3. Fourier-Transform Infrared Spectroscopy

Absorption infrared spectra were recorded using a UV–visible spectrophotometer (U2910, Hitachi, Tokyo, Japan) in attenuated total reflection (ATR) mode. Fourier-transform infrared spectroscopy (FTIR) was performed at room temperature on an FTIR spectrometer (Vertex 80, Bruker, Karlsruhe, Germany) in the range of 750 to 1750 cm^−1^ with an interval of 250 cm^−1^. Each spectrum represented an average of 5 spectra after baseline correction; the absorbance was normalized for uniformity in presentation and analysis. The spectral resolution was 4 cm^−1^.

### 3.4. Determination of Retention Capacity and Pi Release Rates of Innovative Fertilizers

To obtain the Pi retention capacity (encapsulant efficiency) of the microencapsulates, 80 mg of each fertilizer (F0, F1, and F2) was placed inside a bag of dialysis tubes with a pore size of 15,000 kDa (Spectra^®^/Pore cellulose, Wilmington, MA, USA), and these were independently transferred to a glass beaker containing 250 mL deionized water (without stirring) at pH 4 and 6, respectively. Then, the electrical conductivity was measured at 1, 15, 30, 45, 60, 90, 120, and 240 min after immersion. A Corning^®^ conductivity/TDS/temperature meter, benchtop model 441 (New York, NY, USA), was used for this purpose. MicroSiemens (μS) values were used to measure the Pi release rate. The respective calculations were performed according to a calibration curve in which KH_2_PO_4_ was used as a standard; each calibration curve had a coefficient of determination ≥ 0.98. All tests were conducted in triplicate.

### 3.5. Assay to Determine Pi Release Rates of Innovative Fertilizers in Sand–Perlite–Vermiculite Substrate

An assay was performed using an inert substrate placed in pots in order to measure the amount of dissolved Pi that was gradually released from the encapsulated fertilizers into the soil. To perform these assays, a sterile substrate [sand–perlite–vermiculite (2:1:1), autoclaved for 45 min at 121 °C and 20 psi] placed in 7 cm plastic square plant pots was supplemented with specific amounts of the IFs (250 and 500 ppm, respectively). Moreover, the CF and a sand–perlite–vermiculite substrate without fertilizer were used as controls. The pots were maintained at the maximum water-holding capacity (MWHC). For this, they were first irrigated with 15 mL of deionized water; once the MWHC was reached, it was maintained with the daily addition of 3 to 5 mL water (until the initial weight obtained after the first irrigation step was reached), which guaranteed that there were no losses due to leaching. Pots containing the sand–perlite–vermiculite substrate without fertilizer were used as the negative control. At different sampling points (4, 8, and 12 days), the substrate was rinsed using deionized water (25 mL). The recovered leachate was centrifugated (6000 rpm for 15 min), and the supernatant was filtered using a 33 mm diameter sterile syringe filter with a 0.22 µm pore size. As in the release assays performed with water (see above), the amount of dissolved Pi that was gradually released from the encapsulated fertilizers was spectrophotometrically measured by conductivity. During the experiment, the pots were maintained in a plant growth chamber (Caron, Model 7304-50, Marietta, OH, USA) at 22 °C, under a 16:8 h (light–dark) photoperiod and 40–50% relative humidity.

### 3.6. In Vitro and In Vivo Arabidopsis thaliana Growth Bioassays

The effectiveness of the microencapsulated innovative fertilizers (and encapsulant matrices) was evaluated in in vitro assays using the model plant *Arabidopsis thaliana* as a wild type (Col-0) and transgenic line *AtPT2::uidA* [92]. In these transgenic plants, the *uidA* reporter gene [93] under the regulation of the promoter of high-affinity phosphate transporter (AtPT2), can visually indicate (after a histochemical process, as described below) the activity of this phosphate transporter through beta-glucuronidase (GUS) enzymatic activity. These assays allowed us to study plants growing under Pi starvation and determine the changes in the phosphate-transport-related response as the encapsulated Pi was released from the innovative fertilizers. Briefly, the seeds were surface-sterilized with five minutes of exposure to 70% ethanol, followed by 7 min of exposure to 20% commercial bleach. Subsequently, the seeds were washed thrice with sterile distilled water and stored at 4 °C for 48 h. Finally, the seeds were germinated and grown on Petri dishes containing sterile modified Murashige and Skoog medium 0.1× (pH 5.7, 0.5% [*w*/*v*] sucrose and 1% [*w*/*v*] agar), using variable concentrations of KH_2_PO_4_ (also called conventional fertilizer (CF) and supplemented at 0, 8, 32, and 136 ppm) as a Pi source. The effectiveness and influence of the encapsulant matrices used to formulate the IFs were also tested. All formulations (F0, F1, F2, CM0, UMCM1, and UMCM2) were independently mixed in a medium without phosphate before its solidification. In all cases, the seeds were grown in Petri dishes under a photoperiod consisting of 16 h of light and 8 h of darkness and at a temperature of 22 °C in a plant growth cabinet (Percival Scientific, Perry, IA, USA). The plates were placed vertically at an angle of 65° to allow root growth along the agar surface and unimpeded hypocotyl growth into the air.

The innovative fertilizer that yielded the best plant response in the in vitro assays (F2 fertilizer; see Section 2) was also tested in a potting trial (in vivo assay). This experiment was conducted in a greenhouse using 7 cm diameter pots containing 50 g dry substrate. For this purpose, 21-day-old *A. thaliana* seedlings were first grown in vitro under optimal phosphate conditions, namely 136 ppm or 1 mM (considering the additional weight of the encapsulant matrix). They were then transplanted to sterile sand–perlite–vermiculite (2:1:1) supplemented either with 136 ppm of the F2 fertilizer (KH_2_PO_4_ encapsulated with UMCM2) or with the conventional fertilizer (CF; KH_2_PO_4_) only. The same sterile substrate without a fertilizer source was used as a control. The greenhouse temperature was maintained at 26/23 °C (day/night), and relative humidity of 90% was considered during the growing period (matched to the climatic conditions of the study site). Irrigation with deionized water was applied whenever needed to maintain the substrate’s moisture (~80% of field capacity). The plant’s growth characteristics were measured at 15 and 30 days after its transplantation.

It should be noted that, in the in vitro assays, for each evaluated treatment (innovative fertilizers, encapsulant matrices, and positive and negative controls), either the plant growth phenotype or the histochemical staining of the GUS activity (as described above in the Section 2) was uniformly observed in all seedlings used in the different bioassays. This amounted to at least thirty seedlings per treatment, which were grown on three independent Petri dishes with 10 seedlings per dish (three biological replicates). Regarding the in vivo assays, the phenotype described was observed in at least ten seedlings grown in independent pots.

### 3.7. Histochemical Analysis

For the histochemical analysis conducted to visualize the GUS activity, 15-day-old *AtPT2::uidA* seedlings were harvested and incubated overnight at 37 °C in a GUS reaction buffer (0.5 mg/mL of 5-bromo-4-chloro-3-indolyl-b-D-glucuronide in 100 mM sodium phosphate, pH 7). The stained seedlings were cleared with 90% lactic acid or as described previously [94] for each treatment tested (CF, F0, F1, F2, CM0, UMCM1, and UMCM2) at different concentrations (8, 32, and 136 ppm), including the negative control (P^−^). At least 15 transgenic plants were analyzed. For each treatment, an image of a representative plant was captured using Nomarski optics on a Leica DM2500 microscope (Leica, Wetzlar, Germany.

### 3.8. Statistical Analyses

To determine the effects of the different treatments on *A. thaliana* growth, variables such as the length of the principal root (LPR) and the number of lateral roots (LR) were quantified (see the in vitro assay). Fifteen plants (*n* = 15) were considered for each treatment, and in the bar plots, these values were presented as means (±SE). The Shapiro–Wilk test was used to test for normal distribution of the data, and the Bartlett test was used to test for homoscedasticity. One-way ANOVA was used to determine whether the effect of each treatment, either IFs (F0, F1, and F2) or the encapsulating matrices (CM0, UMCM1, and UMCM2), was significant. CF and P deprivation were used as positive and negative controls. When significant differences were observed, post hoc analyses based on Tukey’s test were applied to the data. The InfoStat software v2011 [95] was used for this purpose. Similar statistical analyses were conducted on the results of the in vivo assays, in which the number of plants considered was slightly smaller (*n* = 10).

## 4. Conclusions

This study demonstrates that an environmentally friendly polymer such as chitosan can be modified via ultrasonic treatment to improve certain properties, such as the release rate, mainly when it is used as an encapsulant matrix for anionic compounds such as phosphate fertilizers (e.g., monopotassium phosphate). The modified polymers (UMCM1 and UMCM2) provided the formulated IFs (F1 and F2) with distinctive properties in terms of their capacity as plant growth promoters and/or the delivery of the active compound. These new properties, demonstrated by the F1 and F2 fertilizers, provided advantages in comparison with the conventional fertilizer (CF) and those encapsulated using unmodified chitosan (i.e., F0). The development of these phosphate fertilizers, formulated based on modified chitosan, can be achieved through a relatively low-cost method, such as spray drying. Thus, this process could be easily scalable at an industrial level while avoiding the generation of organic waste at different stages. These formulations should also be easily degradable in the soil, making them ideal for use on a large scale. According to the presented results, modified chitosan behaves similarly to a hydrogel, as it tends to absorb the water in the medium, expanding its chains until reaching an equilibrium. This means that the prolonged release effect is dependent on the medium in which the active compound is released. Finally, we examined the growth-promoting effect, focusing on the F2 formulation. Our results suggest that comparable biomass to those obtained with the “optimal” concentrations (or applications) of the CF can be achieved but with fewer applications. Moreover, using the innovative F2 fertilizer, better yields could be obtained in a reduced amount of time if the typical concentrations (or applications) are used. This is an important feature, especially regarding crops that are of agricultural interest, as well as providing a general solution to the problems of modern agriculture.

## 5. Patents

The method for obtaining fertilizer microparticles encapsulated in modified chitosan has been patented; for details, see Claudia-Anahí Pérez-Torres et al., patent US11639319B2 [36].

## Figures and Tables

**Table 1:** Release models and their correlation rates. This table represents the results of the correlation model established between our data and the theoretical data at a particular time. In each model, the rate constant for polar liquids was used (*K*_0_, *K_H_*, and *K_KP_*, respectively). Values close to 1 obtained for the coefficient of determination (R^2^) are shown in bold font.

Release Model	Equation	Parameters	Fertilizer	R^2^
Zero-order	*Q_t_*: *K*_0_*t*	*Q_t_* is the cumulative amount of KH_2_PO_4_ released at time *t*, *K*_0_ is the zero-order rate constant (*K*_0_ = 0.27), and *t* is the time.	F0	0.763
			F1	0.493
			F2	0.609
Higuchi	*Q_t_*: *K_H_t*^1/2^	*Q_t_* is the cumulative amount of KH_2_PO_4_ released at time *t*, *K_H_* is the zero-order rate constant (*K_H_* = 1.23), and *t* is the time.	F0	0.877
			F1	0.694
			F2	0.793
Korsmeyer–Peppas	*Q_t_*: *K_KP_t^n^*	*Q_t_* is the cumulative amount of KH_2_PO_4_ released at time *t*, *K_KP_* is the Korsmeyer–Peppas rate constant (*K_KP_* = 0.23), and *n* is the release exponent (*n* = 0.85).	F0	**0.975**
			F1	**0.896**
			F2	**0.977**

## Data Availability

Data is contained within the article and Supplementary Material.

## Supplementary Materials

The following supporting information can be downloaded at: https://www.mdpi.com/article/10.3390/plants14040610/s1, Figure S1. Growth of *Arabidopsis thaliana* seedlings in vitro in response to innovative fertilizers and encapsulant matrices. Here, 15-day-old seedlings were grown on Murashige and Skoog (MS) media supplemented with different concentrations (8, 32, and 136 ppm) of either the innovative fertilizers (F0, F1, and F2) or the encapsulated matrices used in their formulation (CM0, UMCM1, and UMCM2). Conventional fertilizer (CF) and a phosphate deprivation treatment (P^−^) were also included as positive and negative controls, respectively. Bars illustrate the effects of these treatments on the main root length (a) and number of lateral roots (b). ANOVA and post hoc Tukey test results (depicted as lowercase letters) represent the significant differences (*p* ≤ 0.05) between the different treatments considering the number of plants sampled (*n* = 15). Figure S2. *AtPT2* gene induction in *Arabidopsis thaliana AtPT2:uidA* line treated with innovative fertilizers or encapsulant matrices. The GUS staining intensity in the plant tissue was quantified using the ImageJ software v1.54 [91], as described in Béziat et al. [96]. The Y-axis shows the percentage of stained area relative to the total tissue area analyzed, either in the leaves (a) or the roots (b). *A. thaliana AtPT2:uidA* seedlings were supplemented with 8, 32, or 136 ppm of the innovative fertilizers (F0, F1, and F2) or the encapsulated matrices used for their formulation (CM0, UMCM1, and UMCM2). Conventional fertilizer (CF) and a phosphate deprivation treatment (P^−^) were also included (positive and negative controls, respectively). Each value is the mean of three biological replicates (with six plants per replicate) ± SE.
