# Supplementary material for: Controlled-Release Phosphorus Fertilizers Manufactured with Chitosan Derivatives: An Effective Alternative for Enhanced Plant Development
Source: Plants (Basel). 2025 Feb 18;14(4):610. doi: 10.3390/plants14040610 (PMC11858907; doi:10.3390/plants14040610)
Supplement: Supplementary file 1 [file plants-14-00610-s001.zip › plants-3384255-supplementary.pdf]

plants

Online supplementary data (Figures S1 and S2)

Controlled-release phosphorus fertilizers manufactured with chitosan derivatives: an effective alternative for enhanced plant development

Eva García-Ilizaliturri, Enrique Ibarra-Laclette, Nicolaza Pariona-Mendoza, Carlos Espinoza-González, Antonio Cárdenas-Flores, José Humberto Valenzuela-Soto, Alan Josué Pérez-Lira and Claudia-Anahí Pérez-Torres

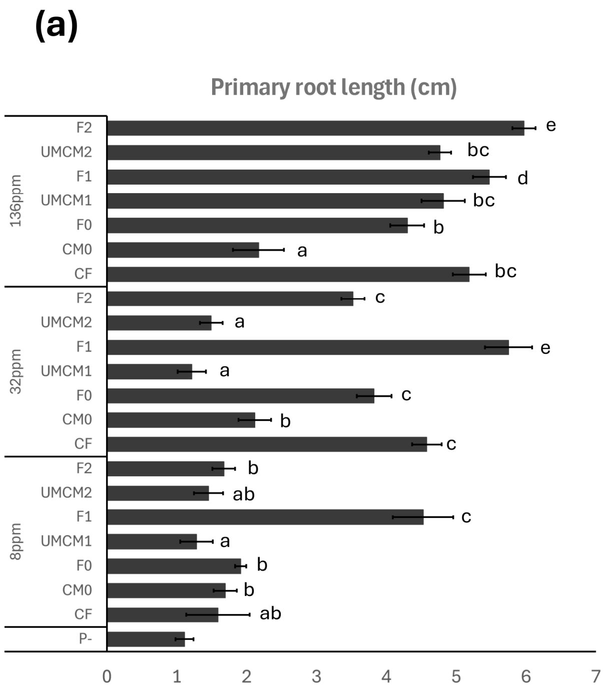

Figure S1. Growth of *Arabidopsis thaliana* seedlings *in vitro* in response to innovative fertilizers and encapsulant matrices. Here, 15-day-old seedlings were grown on Murashige and Skoog (MS) media supplemented with different concentrations (8, 32, and 136 ppm) of either the innovative fertilizers (F0, F1, and F2) or the encapsulated matrices used in their formulation (CM0, UMCM1, and UMCM2). Conventional fertilizer (CF) and a phosphate deprivation treatment (P^-) were also included as positive and negative controls, respectively. Bars illustrate the effects of these treatments on the main root length (**a**) and number of lateral roots (**b**). ANOVA and post hoc Tukey test results (depicted as lowercase letters) represent the significant differences ($p \leq 0.05$) between the different treatments considering the number of plants sampled (n=15).

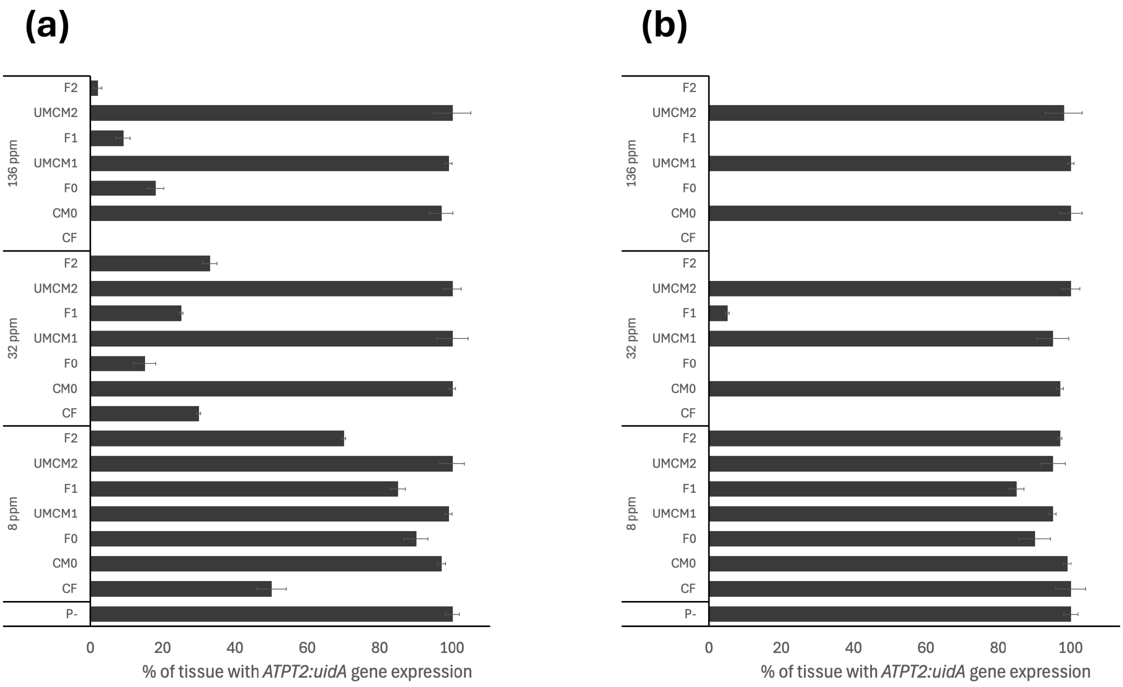

Figure S2. *AtPT2* gene induction in *Arabidopsis thaliana AtPT2:uidA* line treated with innovative fertilizers or encapsulant matrices. The GUS staining intensity in the plant tissue was quantified using the ImageJ software [91], as described in Béziat *et al* [96]. The Y-axis shows the percentage of stained area relative to the total tissue area analyzed, either in the leaves (**a**) or the roots (**b**). *A. thaliana AtPT2:uidA* seedlings were supplemented with 8, 32, or 136 ppm of the innovative fertilizers (F0, F1, and F2) or the encapsulated matrices used for their formulation (CM0, UMCM1, and UMCM2). Conventional fertilizer (CF) and a phosphate deprivation treatment (P-) were also included (positive and negative controls, respectively). Each value is the mean of three biological replicates (with six plants per replicate) ± SE.
